# Entropy-based method for assessing the influence of genetic markers and covariates on hypertension: application to Genetic Analysis Workshop 18 data

**DOI:** 10.1186/1753-6561-8-S1-S97

**Published:** 2014-06-17

**Authors:** Jun Liu, Joseph Beyene

**Affiliations:** 1 Department of Clinical Epidemiology & Biostatistics, McMaster University, Hamilton, Ontario, Canada L8S 4K1

## Abstract

Many complex diseases are related to genetics, and it is of great interest to evaluate the association between single-nucleotide polymorphisms (SNPs) and disease outcome. The association of genetics with outcome can be modified by covariates such as age, sex, smoking status, and membership to the same pedigree. In this paper, we propose a block entropy method to separate two classes of SNPs, for which the association with hypertension is either sensitive or insensitive to the covariates. We also propose a consistency entropy method to further reduce the number of SNPs that might be associated with the outcome. Based on the data provided by the organizers of Genetic Analysis Workshop 18, we calculated the block entropies for six different blocking strategies. Using block entropy and consistency entropy, we identified 230 SNPs on chromosome 9 that are most likely to be associated with the outcome and whose associations with hypertension are sensitive to the covariates.

## Introduction

Hypertension is the leading cause of cardiovascular disease worldwide. In the period 1999 to 2002, 28.6% of the U.S. population had hypertension, and several million people in the world currently have this condition [1]. This disease is often called the "silent killer" because it may not cause symptoms until the patient has sustained serious damage to the arteries, brain, and kidney. With advances in technology, more genetic information is generated, and association studies of genetic variants with disease are routinely investigated. Here we focus on the combined role of genetics and covariates [2] in the development of hypertension.

In this paper, we propose a method to separate two classes of single-nucleotide polymorphisms (SNPs) from all SNPs, for which the associations with hypertension are either sensitive or insensitive to the covariates by calculating the block entropy or block entropy gain of SNPs of people who are split into different blocks according to their covariates. We also propose a method to check the consistency of the proportions of the outcome grouped by the main genotype among the blocks by an entropy method that will help us identify the SNPs that are probably associated with the disease outcome. Based on the combined results of the block entropy and consistency entropy, we can find the SNPs that are most probably associated with the outcome.

We believe that our method is useful for detecting important associations between outcome and genetic markers as well as covariates. For example, if hypertension is associated with a covariate, the proportion of hypertension will be different for different covariate values. In that case, we say that hypertension is sensitive to the covariate. Similarly, if hypertension is associated with a SNP, the proportion of hypertension in different genotypes will be different. Therefore, we say that hypertension is sensitive to the SNP.

## Materials and methods

### Genotype, covariates, and phenotype data

Our methods for investigating the relationship between SNPs and hypertension are based on data provided by the organizers of Genetic Analysis Workshop 18 (GAW18). Genotype data are provided for 11 odd-numbered chromosomes. We use all the SNPs on chromosomes 3 and 9 from genome-wide association studies (GWAS) files, including all people with genotype data. A total of 20 pedigrees are provided with information on father, mother and sex. The phenotype (hypertension) is measured for a maximum of 4 points. In this paper, we use the baseline hypertension status. We include the following covariates at baseline: Sex, Smoke, and Age.

### Block entropy and block entropy gain

Shannon entropy [3] is used as a measure of genetic diversity [4]. For a binary random variable *E *with probability p=Prob{E=1}, Shannon entropy is defined as $-plog_{2}p.$ We define the entropy of  E, which equals entropy(*p*, 1*-p*) in information [5],as $G\left(E\right)=-plog_{2}p-\left(1-p\right)log_{2}\left(1-p\right)$.

The variable  E represents an event in a subset of the complete observations, such as hypertension status in a group of people with a proportion of the outcome *p *in the block of sample. Suppose we have *m *blocks; *n *observations are split into these *m *blocks $B_{1},B_{2},\ldots {\text{B}}_{m}$ with block size of $n_{1},n_{2},\ldots ,n_{m},$ respectively. Block $B_{i}$ (*i *= 1, 2, ..., *m*) has $q_{i}$ observations with the outcome. The observations in one of the blocks $B_{i}$ s (*i *= 1, 2, ..., *m*) are assumed to share something in common in which "similarity" might be defined based on covariates and relatedness using covariates such as age, sex, smoking status, and membership to the same pedigree. Because each block has 3 genotype classes for each SNP, block $B_{i}$ can be classified into 3 groups of observations, $B_{i0},B_{i1},$ and $B_{i2}$, with sizes given by $n_{i0},n_{i1},$ and $n_{i2}$, respectively. All of the observations in $B_{ij}$ have the genotype *j*, for *j *= 0, 1, 2. The group $B_{ij}$ has $q_{ij}$ observations with the outcome. For block $B_{i}$, the entropy is

(1) $$G_{i}=\overset{2}{\underset{j=0}{\sum }}\left(-\frac{q_{ij}}{n_{i}}log_{2}\frac{q_{ij}}{n_{ij}}-\frac{n_{ij}-q_{ij}}{n_{i}}log_{2}\frac{n_{ij}-q_{ij}}{n_{ij}}\right).$$

Therefore, we can get the block entropy to represent the entropy with SNPs.

(2) $$G=\overset{m}{\underset{i=1}{\sum }}\frac{n_{i}}{n}G_{i}=\overset{m}{\underset{i=1}{\sum }}\overset{2}{\underset{j=0}{\sum }}\left(-\frac{q_{ij}}{n}log_{2}\frac{q_{ij}}{n_{ij}}-\frac{n_{ij}-q_{ij}}{n}log_{2}\frac{n_{ij}-q_{ij}}{n_{ij}}\right).$$

We also can get the entropy without considering SNPs:

(3) $$G^{*}=\overset{m}{\underset{i=1}{\sum }}\left(-\frac{q_{i}}{n}log_{2}\frac{q_{i}}{n_{i}}-\frac{n_{i}-q_{i}}{n}log_{2}\frac{n_{i}-q_{i}}{n_{i}}\right).$$

And the block entropy gain (I) is defined as $G_{a}=G^{*}-G$.

If we want to show the gain of the block entropy from information of SNPs without considering the covariates, we can get the block entropy gain (II)

(4) $$G_{a}^{\prime }=\overset{2}{\underset{j=0}{\sum }}\frac{1}{n}\left\{-q_{.j}log_{2}\frac{q_{.j}}{n_{.j}}-\left(n_{.j}-q_{.j}\right)log_{2}\frac{\left(n_{.j}-q_{.j}\right)}{n_{.j}}+\overset{m}{\underset{i=1}{\sum }}\left[q_{ij}log_{2}\frac{q_{ij}}{n_{ij}}+\left(n_{ij}-q_{ij}\right)log_{2}\frac{n_{ij}-q_{ij}}{n_{ij}}\right]\right\},$$

where $n_{.j}$ (*j *= 0, 1, 2 ) is the number of observations with genotype *j *and $q_{.j}$ (*j *= 0, 1, 2) is the number of observations with genotype *j *with the outcome.

Consider that the study population is split according to one attribute, for example,age. We calculate $G^{*}$ the entropy for this attribute without considering SNPs. We then calculate $G^{*}$ for another attribute, say Smoke. If $G^{*}$ is smaller for Age, then it demonstrates that Age has stronger associations with hypertension than does Smoke. We calculate the block entropy, *G*, for the blocks split by Age at each locus. The lower value of G shows that adjusting for Age is informative for the SNP. In contrast, the block entropy gain (I) or (II) goes in the opposite way. The higher the block entropy gain is, the more informative the SNP is.

### Associations of genotypes with covariates

A block $B_{i}$ of size ${\text{n}}_{\text{i}}$ with groups of 3 genotypes is formed according to the values of the covariates. In the block $B_{i}$, the proportion of the associations of a genotype *j *with the outcome is $p_{ij}=\frac{q_{ij}}{n_{ij}}$, and the proportions of the outcome in the block is $p_{i}=\frac{q_{i}}{n_{i}}$. For all the *m *blocks and a genotype *j*, there exists $\varepsilon_{1j},\varepsilon_{2j},\ldots ,\varepsilon_{mj}$ and $\varepsilon_{kj}=0$ for $k=\left\{\text{l}:p_{l}=\text{ma}{\text{x}}_{\text{i}\epsilon \left\{1,2,\ldots ,m\right\}}\left\{p_{i}\right\}\right\}$ such that the following proportion equation is satisfied,that is, $p_{1}:p_{2}:\ldots :p_{m}=\left(p_{1j}+\varepsilon_{1j}\right):\left(p_{2j}+\varepsilon_{2j}\right):\ldots :\left(p_{mj}+\varepsilon_{mj}\right)$. Therefore, $\frac{max_{k\epsilon \left\{1,2,\ldots ,m\right\}}\left\{p_{k}\right\}}{p_{1}}\left(p_{1j}+\varepsilon_{1j}\right)=\frac{max_{k\epsilon \left\{1,2,\ldots ,m\right\}}\left\{p_{k}\right\}}{p_{2}}\left(p_{2j}+\varepsilon_{2j}\right)=\ldots =\frac{max_{k\epsilon \left\{1,2,\ldots ,m\right\}}\left\{p_{k}\right\}}{p_{m}}\left(p_{mj}+\varepsilon_{mj}\right).$ It is clear that if the genotype *j *is fully associated with the covariates and the outcome is fully associated with the covariates, then $\varepsilon_{1j},\varepsilon_{2j},\ldots ,\varepsilon_{mj}=0$. We can say the genotype *j *in this extreme condition is consistent. If there is no relationship between the genotype and the covariates, $\varepsilon_{ij}$ can be any value. Let the size of the block belarge enough to have the blocking effects, say $n_{0}$. We define the standardized proportion of the associations of the genotype *j *with the outcome in block $B_{i}$ as

(5) $$F\left(i,j\right)=\left\{\begin{array}{c} \frac{max_{k\epsilon \left\{1,2,\ldots ,m\right\}}{\left\{\frac{q_{k}}{n_{k}}\right\}}_{n_{k}\geq n_{0}}}{\frac{q_{i}}{n_{i}}}\frac{q_{ij}}{n_{ij}},for\;n_{k}\geq n_{0}\\ \frac{q_{ij}}{n_{ij}},for\;n_{k}<n_{0} \end{array}\right)$$

where $n_{0}$ is a constant based on the overall block sizes.

The standardized proportion of the total associations of a genotype *j *with the outcome at a locus is defined as $F\left(j\right)=\overset{m}{\underset{i=1}{\sum }}\frac{n_{i}}{n}F\left(i,j\right).$

### The consistency of the main genotype

Because a block includes people with 3 genotypes of SNPs, the associations of a genotype *j *with the outcome may have a higher standardized proportion in one block but lower in another block. This genotype is not consistent in the 2 blocks. Although the entropy is low for this SNP, which means that the associations of the SNP and covariates with the outcome are high, the nonconsistency of the genotype across the blocks leads to a weak conclusion that these informative SNPs are related tothe covariates.

Suppose for a SNP at a locus, the standardized proportion of the total associations of a genotype *j *with the outcome is F(*j*). We choose *j *satisfying $\text{F}\left(j\right)=max_{k\epsilon \left\{0,1,2\right\}}\left\{F\left(k\right)\right\}$. We define entropy to check the consistency of the standardized proportion of the associations of this main genotype j.

(6) $$G\left(j\right)=\overset{m}{\underset{i=1}{\sum }}\frac{n_{i}}{n}\left(-\frac{F\left(i,j\right)}{\sum_{i=1}^{m}F\left(i,j\right)}log_{2}\frac{F\left(i,j\right)}{\sum_{i=1}^{m}F\left(i,j\right)}-\frac{\sum_{i=1}^{m}F\left(i,j\right)-F\left(i,j\right)}{\sum_{i=1}^{m}F\left(i,j\right)}log_{2}\frac{\sum_{i=1}^{m}F\left(i,j\right)-F\left(i,j\right)}{\sum_{i=1}^{m}F\left(i,j\right)}\right).$$

The greater the consistency entropy of the main genotype *j *is, the more powerful the consistency of the genotype *j*.

### The block entropy or entropy gain algorithm

We first identify observations with complete genotype data; obtain the values of the covariates Sex, Smoke, and Age as well as classes of pedigree and hypertension at baseline $\text{HT}{\text{N}}_{1}$. Then we choose a blocking strategy, for example, the blocking strategy based on the attribute Age.

We next calculate the block entropy or block entropy gain according to the above blocking strategy at each locus and order the block entropies in ascending order for all loci. The upper *s*% (e.g., 15%) of SNPs in ascending order of the block entropies are those whose associations with the outcome are related to the covariates. The lower *i*% (e.g.,15%) part of loci define the SNPs whose effects are insensitive to the covariates, among which many loci include rare genotypes.

For the upper *s*% part of loci, we find the main genotype *j *and calculate the consistency entropy for the standardized proportion of the associations of the genotype *j *with the outcome. We remove the SNPs with the lower consistency entropies; the SNPs associated with the outcome remain. We can combine some block entropies and consistency entropies for these SNPs to find the important SNPs associated with the outcome.

## Results

### Application to real data

Our first blocking strategy takes all observations as a block. As a crude estimation, the block entropy or entropy gain can classify the SNPs at loci as either associated with the outcome or not associated with the outcome. We next choose the blocking strategy based on a single attribute (Sex, Smoke, Age, and Pedigree) and calculate the block entropy and block entropy gain for all the SNPs, respectively. For the attribute Sex, the people are blocked based on Sex = 1 and Sex = 2. For the attribute Smoke, the people are blocked based on Smoke = 0 and Smoke = 1. For the attribute Age, the people are blocked based on Age < 60 and Age ≥ 60. And for the attribute Pedigree, there are 20 pedigrees splitting the entire sample. Therefore, the people are grouped in 20 blocks to calculate the block entropy and block entropy gain for the attribute Pedigree.

Finally, we choose the blocking strategy based on all the attributes such that all observations are split into the blocks for all the values of these attributes. For example, the people with Sex =1, Smoke = 0,Age< 60, and Pedigree =2 are in one block. The people with the attributes Sex= 1, Smoke = 0, Age ≥ 60, and Pedigree = 3 form another block.

Using the block entropy approach, we classify the SNPs related to the disease outcome as either sensitive or insensitive to the covariates. Here we focus on the SNPs sensitive to the covariates. We also calculate the entropies for checking the consistency of the main genotype among the blocks. The rules are $\text{BE}<V_{BE}$ and $\text{CE}>V_{CE},$ where BE is a block entropy, CE is a consistency entropy, and $V_{BE}$ and $V_{CE}$ are limit values of BE and CE, respectively. For each one of consistency entropies, we remove around 20% of inconsistent SNPs. For CE_Ped, we keep around 40% of SNPs. And $V_{BE}=BE_{min}+k*\left(BE_{max}-BE_{min}\right),$ where $BE_{min}$ is the minimum block entropy based on the covariate and $BE_{max}$ is the maximum one. k = 0.45 for chromosome 9,and k is between 0.5 and 0.7 for chromosome 3.

Selected by the low block entropy with high consistency entropy, we use the rules of BE_All-Attr < 0.36 and BE_No-Attr< 0.67 and CE_Ped > 0.29 and CE_Sex > 0.9 and CE_Smoke > 0.9 and CE_Age > 0.9 (Note:BE_Age is block entropy for blocking strategy based on Age). We choose 230 from 42,178 SNPs in the chromosome 9 GWAS file to be the SNPs sensitive to the covariates. The results shown in Table 1 are 17 SNPs among the 230 loci associated with hypertension that were also found by other researchers in the literature, for example rs774227 [6]; rs6833 is related to hypertension for pregnancy. From Table 1, we know that block entropy of rs6833 for All-Attr is 0.341. It is a small value but not very small compared with all other 42,177 SNPs. We select it because its consistency entropies are high as well.

**Table 1:** Single-nucleotide polymorphisms in chromosome 9 that are sensitive to the covariates

	No-Attr	Sex		Smoke		Age		Ped		All-Attr
						
ID	BE	BE	CE	BE	CE	BE	CE	BE	CE	BE
rs7030214	0.667	0.663	0.982	0.659	0.991	0.567	0.943	0.587	0.295	0.320
rs4962043	0.668	0.665	0.999	0.662	0.999	0.580	0.987	0.593	0.295	0.328
rs6833	0.670	0.667	0.999	0.662	0.991	0.579	0.994	0.597	0.308	0.341
rs489504	0.666	0.657	0.964	0.659	0.958	0.577	0.987	0.597	0.300	0.334
rs3118667	0.665	0.661	0.994	0.659	0.997	0.579	0.993	0.599	0.295	0.353
rs3094375	0.669	0.667	0.999	0.662	0.990	0.581	0.996	0.600	0.294	0.348
rs1752337	0.669	0.663	0.996	0.663	0.992	0.575	0.979	0.601	0.291	0.308
rs652600	0.663	0.661	0.999	0.658	0.996	0.578	0.999	0.604	0.292	0.354
rs3124768	0.668	0.665	0.996	0.662	0.999	0.581	0.996	0.604	0.296	0.358
rs7867300	0.666	0.663	0.999	0.658	0.999	0.581	0.999	0.605	0.292	0.343
rs4877972	0.668	0.665	0.995	0.661	0.998	0.578	0.997	0.605	0.302	0.341
rs10781268	0.669	0.664	0.983	0.659	0.984	0.579	0.997	0.606	0.301	0.342
rs10811664	0.666	0.662	0.999	0.657	0.985	0.573	0.979	0.609	0.309	0.337
rs774227	0.668	0.663	0.960	0.661	0.999	0.574	0.999	0.619	0.293	0.359
rs557749	0.669	0.662	0.999	0.658	0.983	0.576	0.995	0.619	0.306	0.337
rs2422493	0.669	0.665	0.997	0.661	0.999	0.579	0.998	0.611	0.300	0.339
rs2184026	0.669	0.664	0.997	0.661	0.952	0.574	0.998	0.611	0.295	0.346

Note: BE is the block entropy; CE is the entropy for consistency. These 17 SNPs are among the 230 loci associated with hypertension that were also found by other researchers in the literature.

We use the rules ofBE_All-Attr < 0.36 and BE_Pedigree < 0.605 and BE_Age < 0.579 and BE_Sex < 0.667 and BE_No-Attr < 0.677 and CE_Ped > 0.29 and CE_Sex > 0.9 and CE_Smoke > 0.9 and CE_Age > 0.9. We choose 156 from 65,519 SNPs in the chromosome 3 GWAS file to be the SNPs sensitive to the covariates. The results shown in Table 2 are 6 SNPs among the 156 loci associated with hypertension that were also found by other researchers in the literature.

**Table 2:** Single-nucleotide polymorphismsin chromosome 3 that are sensitive to the covariates

	No-Attr	Sex		Smoke		Age		Ped		All-Attr
						
ID	BE	BE	CE	BE	CE	BE	CE	BE	CE	BE
rs6804033	0.669	0.663	0.997	0.659	0.992	0.577	0.979	0.590	0.291	0.341
rs399703	0.669	0.665	0.999	0.659	0.978	0.576	0.997	0.594	0.293	0.338
rs2844347	0.670	0.665	0.999	0.662	0.991	0.577	0.996	0.592	0.295	0.339
rs2555239	0.666	0.662	0.999	0.659	0.999	0.578	0.999	0.595	0.290	0.330
rs1918026	0.663	0.657	0.997	0.654	0.983	0.575	0.996	0.602	0.305	0.349
rs9813198	0.674	0.666	0.988	0.667	0.998	0.577	0.997	0.603	0.292	0.345

Note: BE is the block entropy; CE is the entropy for consistency. These 6 SNPs are among the 156 loci associated with hypertension that were also found by other researchers in the literature.

### Application to simulated phenotype data

Two hundred simulated phenotype data files are provided by GAW18. For each simulated phenotype data, we calculate the block entropy, block entropy gain (II), and consistency entropy for the SNPs under 8 situations. The first one takes all observations as a block to calculate the block entropy. Then we choose the blocking strategy based on Sex, Age, and Pedigree, respectively, in the next 3 situations. The fifth calculates the block entropy with split blocks according to all the values of Sex, Smoke, Age, and Pedigree. The sixth situation uses the blocking strategy of the fifth one plus a consistency entropy for pedigree in that the consistency entropy for the selected SNPs should be bigger than the average of the whole consistency entropies. The seventh situation uses the blocking strategy, splitting the people based on all the values of Sex, Smoke, and Age. The last situation uses the seventh blocking strategy but calculates the block entropy gain (II). We pick the values of the above 8 situations for the SNPs of "functional" genes for the GAW 18 phenotype simulations to see whether they are in the top 5% of the SNPs in the simulated data or not. We count the number of the simulations going into the top 5% for these SNPs of functional genes. The results are summarized in Tables 3 and 4. In Table 4, the number of simulations of rs6442089 going into lowest 5% unadjusted block entropy is 74 in all the 200 simulations. This means rs6442089 is an important SNP without consideration of attributes. The table also shows that it is important for Sex, Age, and Pedigree; rs1060407, rs1131356, and rs2322142 are top SNPs according to our calculations from Table 4. Comparing Tables 3 and 4, we can see that the simulated SNPs in chromosome 9 are less important than those in chromosome 3.

**Table 3:** Counts of "functional"single-nucleotide polymorphismsin the top 5% single-nucleotide polymorphisms in chromosome 9 for the 200 simulations

ID	No-Attr BE	Sex BE	Age BE	Ped BE	All-Attr BE	All-Attr BE+CE	SSA BE	SSA BEG
rs2776859	5	4	4	44	12	18	6	15
rs2182870	3	3	7	12	17	34	23	24
rs1197774	8	5	5	37	32	32	26	31
rs11791740	14	16	21	0	0	0	5	3
rs2230287	64	63	56	1	0	0	34	8

Note. BE is the block entropy; CE is the entropy for consistency. BE+CE is block and consistency entropy; BEG is block entropy gain (II); SSA is Sex, Smoke, and Age.

**Table 4:** Counts of "functional" single-nucleotide polymorphismsin the top 5% single-nucleotide polymorphismsin chromosome 3 for the 200 simulations

ID	No-Attr BE	Sex BE	Age BE	Ped BE	All-Attr BE	All-Attr BE+CE	SSA BE	SSA BEG
rs6442089	74	72	74	66	35	55	57	24
rs1060407	76	66	58	50	18	41	46	13
13rs3772219	2	2	6	20	52	44	9	13
rs1131356	20	28	13	25	60	67	41	29
rs4679394	22	21	6	4	0	0	6	2
rs4683602	10	7	28	7	0	0	16	16
rs16851435	34	34	44	0	0	0	19	14
rs304079	4	6	8	16	38	32	19	21
rs373572	7	4	14	17	16	24	19	28
rs2322142	21	24	33	55	70	34	36	33

Note. BE is the block entropy; CE is the entropy for consistency. BE+CE is block and consistency entropy; BEG is block entropy gain (II); SSA is Sex, Smoke, and Age.

## Discussion

We use the block entropy to classify SNPs whose associations with hypertension are either sensitive or insensitive to covariates. From the calculations based on the data provided by GAW18, we show that entropy-based methods might be useful in separating these two classes of SNPs. The entropy without considering the SNPs can show that different covariates have different associations with hypertension. The associations of the SNPs and the covariates with hypertension are shown in the block entropy. Comparing the ordered block entropies for the entire observations as a block with those for observation blocks by sex, we can see that the blocking strategy by the covariate Sex reduces the block entropy. This means that the attribute Sex together with the SNPs enriches the information of their associations with hypertension. The attribute Age is very important because the block entropies based on Age are much lower than those based on the whole observations in a block without split as the blocking strategy. Clearly,Pedigree is the most informative and complex attribute. It has much impact on the outcome. Different pedigrees affect the outcome differently. Because of the complexity of the pedigrees, the entropy for the consistency among the block of people split based on the pedigrees is small compared with the other covariates.

Results for the simulated data also show the effectiveness of the block entropy, the block entropy gain (II), and the consistency entropy. These methods can be used independently or in combination.

Following a suggestion by a referee and as proof-of-principle, we used a permutation framework to assess statistical significance. The process is computer intensive-- it took 12 days to summarize results of 100 permutations for only 28 of 200 simulated phenotype data. The results from this small-scale permutation showed that the number of true positives detected in Tables 3 and 4 are noteworthy and that the false-positive rate is also unlikely to be high.

## Competing interests

The authors declare that they have no competing interests.

## Authors' contributions

JL designed the overall study, performed all of the data analysis and drafted the manuscript. JB assisted in conceiving the idea, drafting the manuscript, and provided overall supervision of the analysis and write up. Both authors read and approved the final manuscript.
